# Supplementary material for: Evaluating Parental Knowledge and Behaviors Regarding Developmental Toxicants in Jazan, Saudi Arabia Using the Prevention of Toxic Chemicals in the Environment for Children Tool (PRoTECT)
Source: Healthcare (Basel). 2024 Sep 4;12(17):1764. doi: 10.3390/healthcare12171764 (PMC11395152; doi:10.3390/healthcare12171764)
Supplement: Supplementary file 1 [file healthcare-12-01764-s001.zip › healthcare-3156614-supplementary.pdf]

Table S1. Distribution of study participants across selected primary healthcare centers in Jazan region

Health Sectors	Selected PHCCs	Sample size	
		Father	Mothers
Central	Mukatat 5	26	27
	Almaboug	27	26
Western	Sabiah	26	27
	Damad	27	26
Mountain	Balghazi	26	27
	Alaidabi	27	26
Southern	Samitah	26	27
	Ahad Almasriha	27	26
Total		424 participants	

Distribution of participants (n=424) across four health sectors in Jazan. Two primary healthcare centers (PHCs) were randomly selected from each sector. Fathers and mothers were equally represented (n=212 each). PHCCs = primary healthcare centers.

Questionnaire in English

Evaluating Awareness of Toxic Chemicals and Their Impact on Children's Health: A Survey for Parents in Jazan, Saudi Arabia

Before beginning the first section, you should know the following: Developmental disorders in children include the following conditions:

1- Learning difficulties
2- Autism Spectrum Disorder
3- Attention Deficit Hyperactivity Disorder

Section One: Demographics

No.	Question	Answer
1	Do you agree to participate in this survey?	I agree *If the answer is yes, continue the survey. I do not agree *If the answer is no, do not continue the survey.
2	Who is participating?	Father, Mother
3	Age	• 18-20, • 21-25, • 26-30, • 31-35, • 36-40, • 41-45, • 46-50, • 51-55, • 56-60, • 61-65, More than 56 years
4	What is your educational level?	Did not attend school, Elementary, Intermediate, High school, University, Postgraduate studies
5	What is the family's monthly income in Saudi Riyals?	• No income, • Less than 4,000 SAR, • From 4,000 to less than 8,000 SAR, • From 8,000 to less than 12,000 SAR, • From 12,000 to less than 16,000 SAR, • From 16,000 to less than 20,000 SAR, • From 20,000 to less than 24,000 SAR, • 24,000 or more
6	Where do you live?	• Village, • City, Mountainous area
7	Name of the primary health care center	
8	Type of residence	• Villa, • Floor in a villa, • Apartment, • Traditional house, • Floor in a traditional house, • Other (Specify).........
9	Social status	• Married, • Divorced, • Widow/Widower
10	Employment status	• Employed for wage, • Works without pay (for the family), • Self-employed and employs others, • Self-employed (does not employ others), • Looking for work and has never worked before, • Looking for work and has worked before, • In education, • In training, • Housewife/Househusband or family responsibilities, • Retired or elderly, • Other (Specify)
11	What sector do you/they work in?	• Government sector, • Public sector institutions, • Private sector for local institutions, • Private sector for foreign institutions, • Agricultural sector, • Domestic labor sector, • Non-profit organizations, • International organizations or foreign embassies, • Another sector (Specify) ______
12	Number of children?	Write the number
13	Do you work in the health sector?	Yes, No
14	Does any other family member	Yes, No

	work in the health sector?	
15	Do you have children who suffer from learning difficulties?	Yes, No, Not sure
16	Do you have children who suffer from autism?	Yes, No, Not sure
17	Do you have children who suffer from attention deficit hyperactivity disorder?	Yes, No, Not sure

Before beginning the second section, you should know the following: Toxic chemicals: These can be found in our homes, air, and water, and can be harmful to our health. They are divided into several types:

1- Petroleum-related pollutants such as gasoline, toluene, ethylbenzene, and xylene
2- 2- Heavy metals such as lead, mercury, arsenic, and cadmium
3- 3- Agricultural chemicals such as pesticides and fertilizers
4- 4- Waste-related pollutants

Developmental disorders in children include the following conditions:

1- Learning difficulties
2- 2- Autism Spectrum Disorder
3- 3- Attention Deficit Hyperactivity Disorder

Section Two: Survey to Measure the Community's Awareness of the Impact of Toxic Chemicals on Children in the Surrounding Environment

Part One: Pregnancy Complications

Question	Strongly Disagree	Somewhat Disagree	Neither Agree nor Disagree	Somewhat Agree	Strongly Agree	No.
Reducing exposure to toxic chemicals during pregnancy and early childhood can help reduce the risk of a child developing disorders such as ADHD or autism.						1
Exposure to toxic chemicals during pregnancy can increase the risk of a child developing developmental disorders.						2
Pregnant women are highly exposed to toxic chemicals.						3

Part Two: Expected Dangers on Children

Question	Strongly Disagree	Somewhat Disagree	Neither Agree nor Disagree	Somewhat Agree	Strongly Agree	No.
The daily toxic chemicals in our lives, such as air pollution or lead in drinking water, can increase the risk of a child developing conditions like ADHD or autism.						4
Toxic chemicals are generally more harmful to children and infants than to adults.						5
Children will benefit more from regulating and reducing toxic chemicals (preventing injury) in developmental disorders than from (treating) these conditions.						6

Part Three: Required Public Policies

Question	Strongly Disagree	Somewhat Disagree	Neither Agree nor Disagree	Somewhat Agree	Strongly Agree	No.
Most countries in the world invest similar amounts in preventing developmental conditions as they do in treating these conditions.						7
We have effective legislation to ensure that food and personal care products do not contain harmful levels of toxic chemicals.						8
When it comes to addressing developmental disorders affecting children, countries spend money managing and treating these conditions. I believe that more should be spent researching ways to prevent children from suffering from these conditions.						9
We should enhance our programs and policies to ensure that consumer products do not contain the toxic chemicals that harm our children.						10

Part Four: Health Promotion

Question	Strongly Disagree	Somewhat Disagree	Neither Agree nor Disagree	Somewhat Agree	Strongly Agree	No.
If toxic chemicals threatened my family's health, my doctor or healthcare provider should inform me about it.						11
I am interested in learning more about how to reduce children's exposure to toxic chemicals.						12

Among all sources of information on the health effects of toxic chemicals, I trust the information provided by the scientists who study them.						13

Part Five: Protection from Toxic Chemicals in Children

**Question**	**Strongly Disagree**	**Somewhat Disagree**	**Neither Agree nor Disagree**	**Somewhat Agree**	**Strongly Agree**	**No.**
All parents have equal opportunities to protect their children from toxic chemicals such as pesticides or heavy metals, regardless of income level, race and origin, or where they live.						14
If I knew how to reduce children's exposure to toxic chemicals, I would definitely try to do so.						15
I try to buy products that do not contain toxic chemicals that could harm my family.						16
I am concerned that my family is being exposed to toxic chemicals.						17
I trust that most companies manufacture products that do not contain harmful levels of toxic chemicals.						18

الاستبيان باللغة العربية

تقييم التوعية بالمواد الكيميائية السامة وتأثيراتها على صحة الأطفال: استبيان للأهالي في جازان، المملكة العربية السعودية

قبل البدء في القسم الأول عليك أن تكون أبا أو تكوني أماً تعرف المقصود بالاتي:

اضطرابات النمو لدى الأطفال تشمل الحالات التالية:

1- صعوبات التعلم
2- اضطراب طيف التوحد
3- اضطراب تشتت الإنتباه وفرط الحركة

القسم الأول: البيانات الديموغرافية

م	السؤال	الإجابة
1	هل توافق على المشاركة في هذا الاستبيان؟	أوافق *إذا كانت الإجابة بنعم يكمل الاستبيان<br>لا أوافق * إذا كانت بلا فلا يكمل الاستبيان
2	المشارك؟	الأب<br>الأم
3	العمر	• 18-20<br>• 21-25<br>• 26-30<br>• 31-35<br>• 36-40<br>• 41-45<br>• 46-50<br>• 51-55<br>• 56-60<br>• 61-65<br>أكثر من 65 سنة
4	المستوى التعليمي؟	لم التحق بالمدرسة<br>ابتدائي<br>متوسط<br>ثانوي<br>جامعي<br>دراسات عليا
5	الدخل الشهري للأسرة بالريال السعودي	• **لا يوجد دخل**<br>• **أقل من 4 آلاف ريال سعودي**<br>• **من 4 آلاف إلى أقل من 8 آلاف ريال سعودي**<br>• **من 8 آلاف إلى أقل من 12 ألف ريال سعودي**<br>• **من 12 ألف إلى أقل من 16 ألف ريال سعودي**

		• **من 16 ألف إلى أقل من 20 ألف ريال سعودي**<br>• **من 20 ألف الى أقل من 24 ألف ريال سعودي**<br>• **24 ألف فأكثر**
**6**	**مكان الإقامة**	• **القرية**<br>• **المدينة**<br>**منطقة جبلية**
**7**	**اسم مركز الرعاية الصحية الأولية**	
**8**	**نوع المسكن**	• فيلا<br>• دور في فيلا<br>• شقة<br>• منزل شعبي<br>• دور في منزل شعبي<br>• أخرى. اذكرذلك.........
**9**	**الحالة الإجتماعية**	• متزوج/ متزوجة<br>• مطلق/مطلقة<br>• أرمل/أرملة
**10**	**الحالة العملية**	• مشتغل/ة بأجر<br>• مشتغل/ة بدون أجر (لدى الأسرة(<br>• صاحب/ة عمل يوظف<br>• يعمل/تعمل لحسابه/ا (لا يوظف(<br>• يبحث/تبحث عن عمل ولم يسبق له/ها العمل<br>• يبحث /تبحث عن عمل وسبق له/ها العمل<br>• ملتحق/ة بالتعليم<br>• ملتحق/ة بالتدريب<br>• متفرغ/ة لأعمال المنزل أو للمسؤوليات العائلية<br>• متقاعد/ة أو كبير/ة سن<br>• أخرى. اذكرها
**11**	**ماهو القطاع الذي تعمل/ين فيه؟**	• القطاع الحكومي<br>• مؤسسات القطاع العام<br>• القطاع الخاص للمؤسسات المحلية<br>• القطاع الخاص للمؤسسات الأجنبية<br>• القطاع الزراعي<br>• قطاع العمالة المنزلية<br>• المنظمات غير الربحية

		• المنظمات الدولية أو السفارات الأجنبية<br>• قطاع آخر (اذكره______)
12	عدد الأطفال؟	اكتب العدد رقماً
13	هل تعمل/ي في المجال الصحي؟	نعم<br>لا
14	هل يعمل أحد أفراد الأسرة الآخرين في المجال الصحي؟	نعم<br>لا
15	هل لديك/لديكي أطفال يعانون من اضطرابات صعوبات التعلم	نعم<br>لا<br>لست متأكداً
16	هل لديك/لديكي أطفال يعانون من اضطراب التوحد	نعم<br>لا<br>لست متأكداً
17	هل لديك/لديكي أطفال يعانون من اضطراب تشتت الإنتباه وفرط الحركة	نعم<br>لا<br>لست متأكداً

قبل البدء في القسم الثاني عليك أن تعرف المقصود بالاتي:

المواد الكيميائية السامة: التي يمكن العثور عليها في منازلنا والهواء والماء، وقد تكون ضارة بصحتنا وهي تنقسم لعدة أنواع:

1 - **الملوثات المتعلقة بالبترول** مثل البنزين والتولوين والإيثيل بنزين والزايلين

2 - **المعادن الثقيلة** مثل الرصاص والزئبق والزرنيخ والكادميوم

3 - **الكيماويات الزراعية مثل** المبيدات والأسمدة

4 - **الملوثات المتعلقة بالنفايات**

اضطرابات النمو لدى الأطفال تشمل الحالات التالية:

1 - صعوبات التعلم

2 - اضطراب طيف التوحد

3 - اضطراب تشتت الإنتباه وفرط الحركة

القسم الثاني: استبيان قياس مدى وعي المجتمع تجاه تأثير المواد الكيميائية السمية بين الأطفال في البيئة المحيطة

م	السؤال	موافق بشدة	موافق إلى حد ما	لا أوافق ولا أعارض	أعارض إلى حد ما	أعارض بشدة
**الجزء الأول: مضاعفات الحمل**						
1	**يمكن أن يساعد تقليل التعرض للمواد الكيميائية السامة خلال الحمل وفي مرحلة الطفولة المبكرة في خفض خطر إصابة الطفل باضطرابات النمو مثل فرط الحركة وتشتت الانتباه أو التوحد.**					
2	**التعرض للمواد الكيميائية السامة خلال الحمل يمكن أن يزيد من خطر إصابة الطفل باضطراب النمو.**					
3	**النساء الحوامل يتعرضن بشدة للمواد الكيميائية السامة.**					
**الجزء الثاني: الأخطار المتوقعة على الأطفال**						
4	**يمكن أن تزيد المواد الكيميائية السامة في حياتنا اليومية، مثل تلوث الهواء أو الرصاص في مياه الشرب، من خطر تطور الطفل لحالات مثل فرط الحركة وتشتت الانتباه أو التوحد.**					

5	المواد الكيميائية السامة عموما أكثر ضررا للأطفال والرضع منها للبالغين.					
6	سيستفيد الأطفال من تنظيم وتقليل المواد الكيميائية السامة في (منع الإصابة) باضطرابات النمو أكثر من (علاج) هذه الحالات.					
	**الجزء الثالث: السياسات العامة المطلوبة**					
7	معظم الدول في العالم تستثمر مبالغ متقاربة لمنع الحالات التنموية كما تنفق لعلاج هذه الحالات.					
8	لدينا تشريعات فعالة لضمان ألا تحتوي الأغذية ومنتجات العناية الشخصية على مستويات ضارة من المواد الكيميائية السامة.					
9	عندما يتعلق الأمر بمعالجة اضطرابات النمو التي تؤثر على الأطفال، تنفق الدول أموالاً على إدارة وعلاج هذه الحالات. أعتقد أنه يجب يُنفق المزيد للبحث عن طرق لمنع الأطفال من الإصابة من هذه الحالات.					
10	يجب أن نعزز برامجنا وسياستنا للتأكد من أن المنتجات الاستهلاكية لا تحتوي على المواد الكيميائية السامة التي تضر أطفالنا					

الجزء الرابع: التعزيز الصحي						
11	لو كانت المواد الكيميائية السامة تهدد صحة عائلتي، كان يجب أن يخبرني الطبيب البيطري، أو الطبيب، أو مقدم الرعاية الصحية عن ذلك.					
12	أرغب في معرفة المزيد حول كيفية تقليل تعرض الأطفال للمواد الكيميائية السامة					
13	من بين جميع مصادر المعلومات حول التأثيرات الصحية للمواد الكيميائية السامة، أثق في المعلومات المقدمة من العلماء الذين يدرسونها.					
الجزء الخامس: الوقاية من المواد الكيميائية السامة لدى الأطفال						
14	لدى جميع الآباء فرص متساوية لحماية أطفالهم من المواد الكيميائية السامة مثل المبيدات الحشرية أو المعادن الثقيلة، بغض النظر عن مستوى الدخل، العرق والأصل، أو المكان الذي يعيشون فيه.					
15	إذا كنت أعرف كيف أقلل تعرض الأطفال للمواد الكيميائية السامة، سأحاول القيام بذلك حتماً.					

16	أحاول شراء المنتجات التي لا تحتوي على المواد الكيميائية السامة التي قد تضر بعائلتي.					
17	أنا قلق من أن تتعرض عائلتي للمواد الكيميائية السامة.					
18	أثق في أن معظم الشركات تصنع منتجات لا تحتوي على مستويات ضارة من المواد الكيميائية السامة.					
